# Toxicology of paraquat and pharmacology of the protective effect of 5-hydroxy-1-methylhydantoin on lung injury caused by paraquat based on metabolomics

**DOI:** 10.1038/s41598-020-58599-y

**Published:** 2020-02-04

**Authors:** Lina Gao, Huiya Yuan, Enyu Xu, Junting Liu

**Affiliations:** School of Forensic Medicine, China Medical University, Liaoning, 110014 China

**Keywords:** Metabolomics, Metabolomics, Molecular medicine

## Abstract

Paraquat (PQ) is a non-selective herbicide and is exceedingly toxic to humans. The mechanism of PQ toxicity is very complex and has not been clearly defined. There is no specific antidote for PQ poisoning. 5-hydroxy-1-methylhydantoin (HMH) is an intrinsic antioxidant and can protect against renal damage caused by PQ. The mechanism of PQ toxicology and the possible effects of HMH on PQ-induced lung injury were determined in this study. It was found that PQ decreased superoxide dismutase (SOD) activity and elevated the level of malondialdehyde (MDA), while HMH elevated SOD activity and decreased the level of MDA. Based on metabolomics, the citrate cycle, glutathione metabolism, taurine and hypotaurine metabolism, regulation of lipolysis in adipocytes, inflammatory mediator regulation of TRP channels, purine and pyrimidine metabolism, aldosterone synthesis and secretion, and phenylalanine metabolism were changed in the PQ group. Compared with the PQ group, the levels of N-acetyl-l-aspartic acid, L-glutamic acid, L-aspartic acid, mesaconic acid, adenosine 5′ monophosphate, methylmalonic acid, cytidine, phosphonoacetic acid, hypotaurine, glutathione (reduced) and cysteinylglycine increased, while the levels of corticosterone, xanthine, citric acid, prostaglandin G2, 4-pyridoxic acid and succinyl proline decreased in the HMH group. These metabolites revealed that HMH can alleviate inflammation caused by PQ and elevate the activity of intrinsic antioxidants. In conclusion, our results revealed PQ toxicology and the pharmacology underlying the protective effect of HMH on lung injury due to PQ. Toxicity caused by PQ results in lipid peroxidation and an increase in reactive oxygen species (ROS), nitric oxide (NO), damage to the biliary system, gastrointestinal system and nervous system, in addition to lungs, kidneys, and the liver. HMH is a good antioxidant and protects against lung injury caused by PQ. In summary, HMH efficiently reduced PQ-induced lung injury in mice.

## Introduction

**Figure 1:**
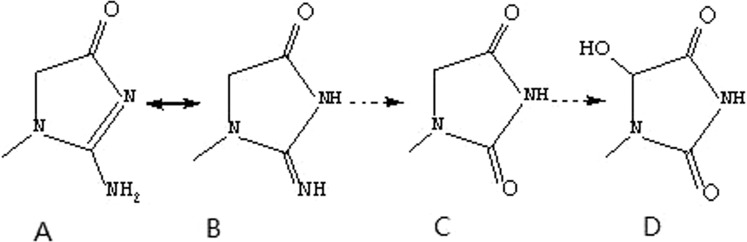
The metabolic pathway of creatinine (**A**. creatinine; **B**. geometric isomer of creatinine; **C**. 1-methylhydantoin **D**. HMH).

Paraquat (PQ, 1,1′-dimethyl-4-4′-bipyridinium dichloride) is a highly toxic quaternary ammonium herbicide widely used in agriculture. The mortality rate of PQ poisoning is as high as 60–80%, mainly due to acute lung injury and progressive pulmonary fibrosis^1–3^. Moreover, there is no specific antidote for PQ poisoning. Following PQ poisoning, the lungs are the main target organs, and the redox reaction occurs after the uptake of PQ in the lungs, which interferes with mitochondrial electron transfer, generates a large number of oxygen free radicals, and induces lipid peroxidation injury^2^. PQ enters the body and is excreted in the form of a prototype in the kidney, where the concentration is highest, resulting in impaired renal function. PQ cannot be excreted normally and further accumulates in the body; thus, involving other organs such as the liver, heart and lung, resulting in multiple organ failure^2^. Creatinine is a degradation product of creatine, which is degraded at a constant rate. When renal function is impaired, large amounts of creatinine are accumulated and metabolized to produce 1-methylhydantoin and 5-hydroxy-1-methylhydantoin (HMH)^4–8^ (as shown in Fig. 1).

High-dose long-term antioxidants are the optimal treatment strategy for improving the survival rate in high-dose PQ poisoning^9^, and a large number of cell and animal experiments have confirmed the effectiveness of exogenous antioxidative therapy^10–12^. HMH is an intrinsic antioxidant and can eliminate hydroxyl radicals^4–8^. Previous studies^13,14^ have shown that HMH protects against PQ-induced kidney damage as it is oxidation resistant.

Metabolomics aims to gather as much information on low-molecule metabolites in biological systems as possible, provide an insight into the cell status, and describe the actual health status of organisms^15–17^. It has been demonstrated to be a promising and powerful approach in studying the influence of disease, applied treatment or diet on the endogenous metabolic state of organisms.

In this study, based on metabolomics and the detection of malondialdehyde (MDA) and superoxide dismutase (SOD), we assessed the protective mechanism of HMH against lung injury induced by PQ poisoning and supplement the PQ toxic mechanism.

## Materials and Methods

### Chemical reagents

HMH was purchased from Shanghai Yanyi Biotechnology Co., Ltd. (Shanghai, China), PQ was from Shanghai Macklin Biotechnology Co., Ltd. (Shanghai, China), the BCA (Bicinchoninic acid) protein detection kit, and SOD total activity detection kit were from Beyotime Biological Reagent Co., Ltd. (Shanghai, China). The malondialdehyde detection kit was obtained from BestBio Biological Reagent Co., Ltd (Shanghai, China). Methanol, water, acetonitrile (ACN) and formic acid (FA) were all liquid chromatography-mass spectrometry (LC-MS) grade, purchased from Thermo Fisher Technology Co., Ltd. (Waltham, MA, USA). LC-MS/MS testing was conducted by Novogene Co., Ltd. (Beijing, China).

### Animal experiments and sample collection

In this study, 4-week old, specific pathogen-free grade Kunming mice (n = 30) weighing 30 ± 2 g were selected. The animals were obtained from Liaoning Changsheng Biotechnology Co., Ltd. The mice were randomly divided into the control group, PQ poisoning group and HMH group, with 10 mice in each group. The PQ poisoning group received PQ 20 mg/kg by gavage. The control group received the same amount of normal saline. The HMH group received an intraperitoneal injection of 100 mg/kg HMH after 20 mg/kg PQ. The HMH group was treated with an intraperitoneal injection at the same time every day for 5 consecutive days. Serum was collected from the mice on the 6^th^ day. All mice were euthanized on the 6^th^ day and lung tissue samples were collected. On the day of sacrifice, the animals were euthanized in accordance with the guidelines of the National Institutes of Health ARAC on the use of carbon dioxide for the euthanasia of rodents. The animal treatment method used in this experiment conforms to the animal ethical standards and has been approved by the ethics center of China Medical University (approval No. 2018072).

### Hematoxylin and eosin staining

After overnight fixation in 10% neutral-buffered formalin, the lungs were dehydrated in alcohol and then embedded in paraffin. Paraffin sections were prepared and stained using standard H&E staining methods^18^.

### Metabolite extraction

Lung tissues (100 mg) were ground with liquid nitrogen and a 100 µL homogenate was resuspended in precooled 100% methanol (−20 °C), followed by vortexing^18^. The samples were incubated at −20 °C for 60 min, then centrifuged at 14000 g, 4 °C for 15 min. The supernatant was transferred to a clean microcentrifuge tube, which was dried in a vacuum in the centrifugal evaporator. The dried metabolite pellets were redissolved with 80% methanol and analyzed by LC-MS/MS^18^.

### LC-MS/MS analysis

LC-MS/MS analysis was performed using the Vanquish UPLC system (Thermo Fisher) and the Orbitrap Q Exactive HF-X mass spectrometer (Thermo Fisher) operating in data-dependent acquisition (DDA) mode. The sample was injected into an Accucore HILIC column (100 × 2.1 mm, 2.6 µm) using a 20-min linear gradient at a flow rate of 0.3 mL/min. The positive polarity mode eluents were eluent A (0.1% FA in 95% ACN, 10 mM ammonium acetate) and eluent B (0.1% FA in 95% ACN, 10 mM ammonium acetate). The negative mode eluents were eluent A (95% ACN, 10 mM ammonium acetate, pH 9.0) and eluent B (50% ACN, 10 mM ammonium acetate, pH 9.0). The solvent gradient was set as follows: 2% B, 1 min; 2–50% B, 16.5 min; 50–2% B, 2.5 min^18^. The MS condition was as follows: Q-exactive HF-X mass spectrometer was selected in the m/z 100–1500 scanning range, and the MS/MS scan was used for a data-dependent full scan. The Q-exactive HF-X mass spectrometer was operated in positive/negative polarity mode with spray voltage of 3.2 kV, capillary temperature of 320 °C, sheath gas flow of 35 arb and aux gas flow of 10 arb.

### Untargeted metabolomics analysis

After metabolic information collection and data preprocessing, the resulting matrix was imported into SIMACA-P (Umetrics, Sweden version 13.0) for unsupervised principal component analysis (PCA) and supervised partial least squares discriminant analysis (PLS-DA)^19^. Identification of metabolites has a variable influence on the projection (VIP) graphs (99% confidence)^19^. For each multivariate model, the calculated R2 value reflects goodness of fit^19^. The parameter Q2 of PLS-DA represents the predictive ability of the model^19^. Q2 close to 0.5 reflects a good model.

Differential variables related to PQ toxicity and HMH pharmacology were monitored as follows: Firstly, the VIP value should be greater than 1.0. Secondly, in order to reduce the possibility of false positives, the adjusted p-value was used for the non-parametric Mann-Whitney U test (PASW Statistics 19, SPSS Inc., Chicago, IL, USA), and the p-value should be less than 0.05^20^. Thirdly, PASW statistic 19 was used to calculate the value of AUC-ROC curve (the area under the receiver operating characteristic). When AUC-ROC was less than or equal to 0.75, the variable was discarded. In addition, when AUC-ROC is greater than 0.9, the classification performance is better^21^. Heat maps of metabolites were drawn from multiple experimental views (version 4.9.0). The changes of metabolites in each group were evaluated by the volcano map. The Kyoto Encyclopedia of Genes and Genome (KEGG) database was used for enrichment analysis and pathway analysis of differential metabolites.

### Data quality evaluation of untargeted metabolomics analysis

Quality control samples (QCs) were obtained by collecting an equal amount of mixture from each lung homogenate sample. A consistency analysis between QCs and actual samples was carried out. Before the batch analysis, the 5 QCs were first tested to stabilize the analysis system and remove the acquired data before data processing. All QCs were used to monitor the robustness of sample preparation and the stability of instrumental analysis by analyzing batch random inserts. During the whole instrumental analysis process, all lung samples were analyzed randomly to avoid inter-batch differences^22^. In order to evaluate the overfitting of the model, 200 permutation tests were performed in the PLS-DA model^20^.

### Ethics approval and consent to participate

All experimental procedures about animal treatment and sample collection were conducted according to the Institutional Animal Care Guidelines and were approved as ethical by the Administration Committee of Experimental Animals at the Laboratory Animal Center of China Medical University.

## Results

### H&E staining

**Figure 2:**
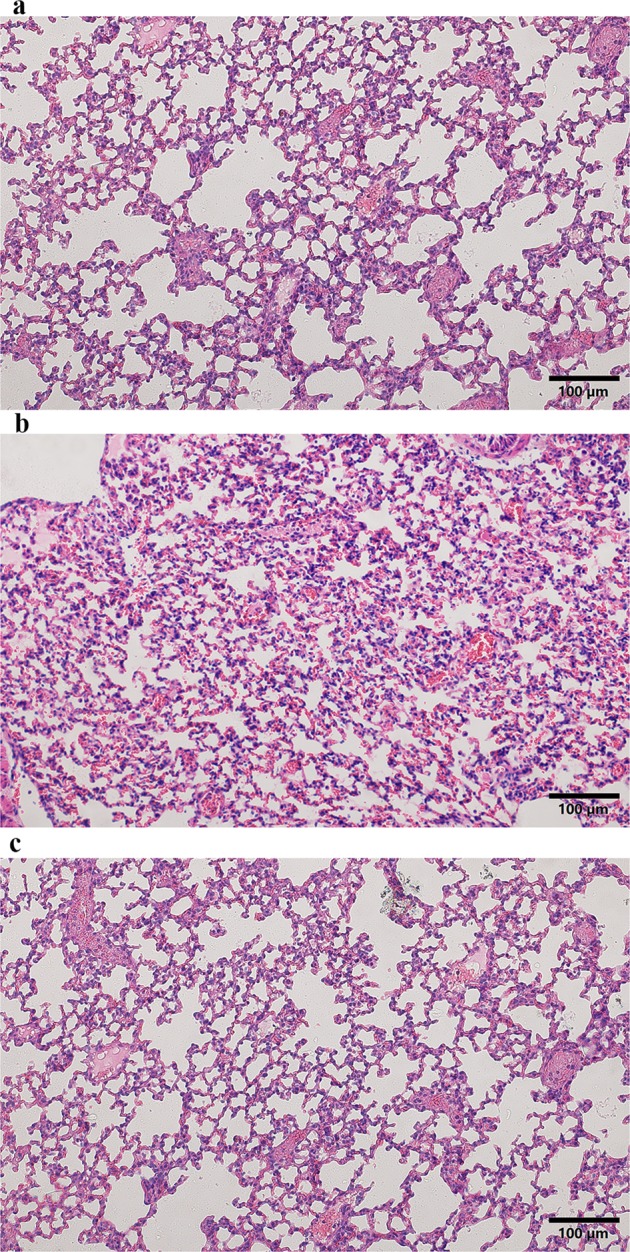
Representative images of H&E staining of lung tissue in the different groups (magnification, 200×), (**a**) the control group, the alveolar structure of the control group was intact, and there was no edema in the alveolar wall and no inflammatory cell infiltration in the lung parenchyma; (**b**) the PQ group, a large number of inflammatory cells infiltrated, with obvious bleeding and clear membrane formation in the alveolar cavity; (**c**) the HMH group, The alveolar structure of the HMH group was slightly damaged with a small amount of inflammatory cells exudation and hemorrhage.

In addition to macroscopic indicators, microscopic morphology was significantly changed between different groups. In the control group, the alveolar structure of the control group was intact, and there was no edema in the alveolar wall and no inflammatory cell infiltration in the lung parenchyma. In the PQ group, abundant inflammatory cell infiltration was observed, with obvious bleeding and clear membrane formation in the alveolar cavity. The alveolar structure in the HMH group was slightly damaged with a small amount of inflammatory cell exudation and hemorrhage, as shown in Fig. 2.

### SOD activity and MDA content

Compared with the control group, the content of MDA was significantly increased and SOD activity in lung tissues was decreased in the PQ poisoning group (MDA (mol/mgprot) 0.54 ± 0.06 vs. 0.13 ± 0.05, SOD (U/mgprot): 172.2 ± 2.54 vs. 272.8 ± 2.54). Compared with the control group, the level of MDA was increased, while SOD activity was significantly decreased in the HMH group (MDA (mol/mgprot) 0.29 ± 0.10 vs. 0.13 ± 0.05, SOD (U/mgprot): 179.7 ± 8.37 vs. 272.8 ± 2.54). Compared with the PQ poisoning group, SOD activity was increased (U/mgprot): 179.7 ± 8.37 vs. 172.2 ± 2.54), and the content of MDA decreased (MDA (μmol/mgprot): 0.29 ± 0.10 vs. 0.54 ± 0.06) in the HMH group as shown in Table 1.

**Table 1:** The level of MDA and the SOD activity in all groups.

	groups	SOD (U/mgprot)	MDA (μmol/mgprot)
	NS	272.8 ± 2.54	0.13 ± 0.05
	HMH	179.7 ± 8.37	0.29 ± 0.10
	PQ	172.2 ± 2.54	0.54 ± 0.06
Pvalue	NS:PQ	3.74E-12**	3.25E-25**
	NS:HMH	0.00024**	1.05E-17**
	HMH:PQ	3.28E-06**	0.014*

Nonparametric Mann–Whitney U test. *p < 0.05; **p < 0.001.

### Sample quality control

The Pearson correlation coefficient between QC samples was calculated based on the peak area value. The higher the correlation of QC samples (R2 is closer to 1), the better the stability of the whole detection process and the higher the data quality. The correlation of QC samples is shown in Supplementary Fig. 3.

### The metabolic pattern in the PQ poisoning group and the control group

#### The PCA and PLS-DA results in the PQ poisoning group and the control group

**Figure 3:**
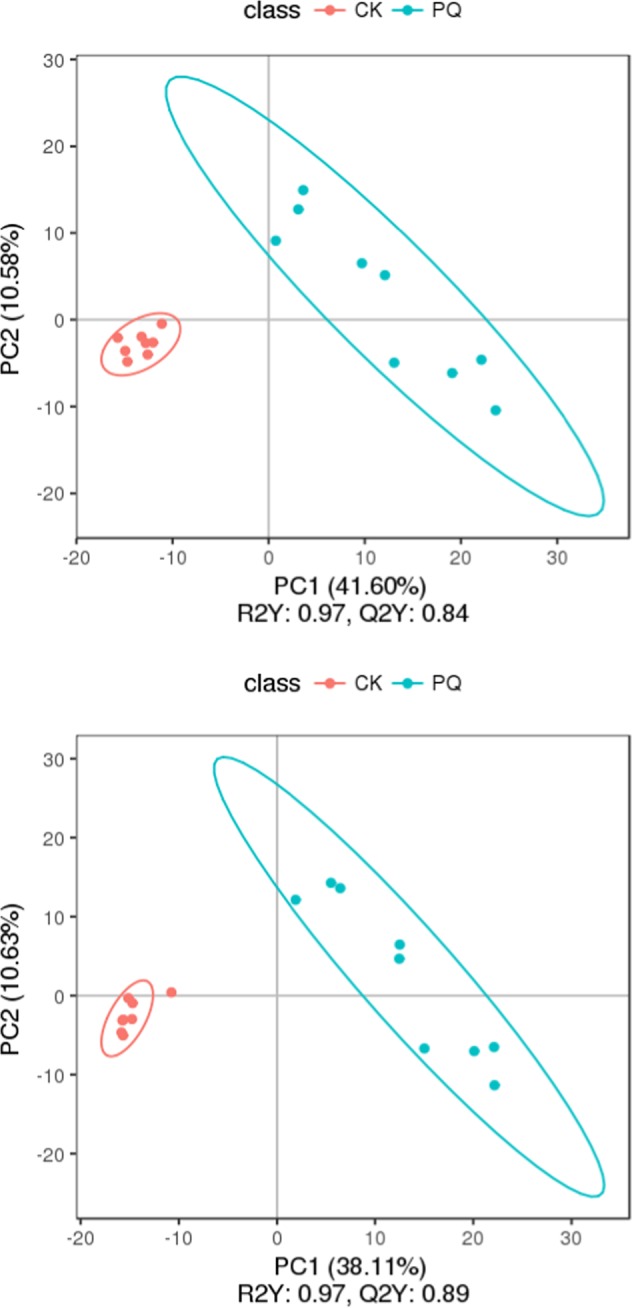
The PLS-DA, the upper figure was obtained in the negative polarity mode, the bottom figure was obtained in the positive polarity mode. Score plot from partial least squares discriminant analysis of the paraquat group and the control group. Each data point represents a function of the entire spectral profile of each subject (n = 19). Partial least squares discriminant analysis showed a clear separation between the 2 groups with acceptable goodness of fit (R^2^ = 0.97) and predictive power (Q^2^ = 0.84 or 0.89).

PCA provided a satisfactory separation of data between the PQ poisoning group and the control group as shown in Supplementary Fig. 1. The potential constituents were screened by PLS-DA as shown in Fig. 3, which revealed the differences in lung tissue in the two groups^16,17^. The PLS-DA scores plot showed very good discrimination between the PQ poisoning group and the control group.

#### The volcano maps of differential metabolites

**Figure 4:**
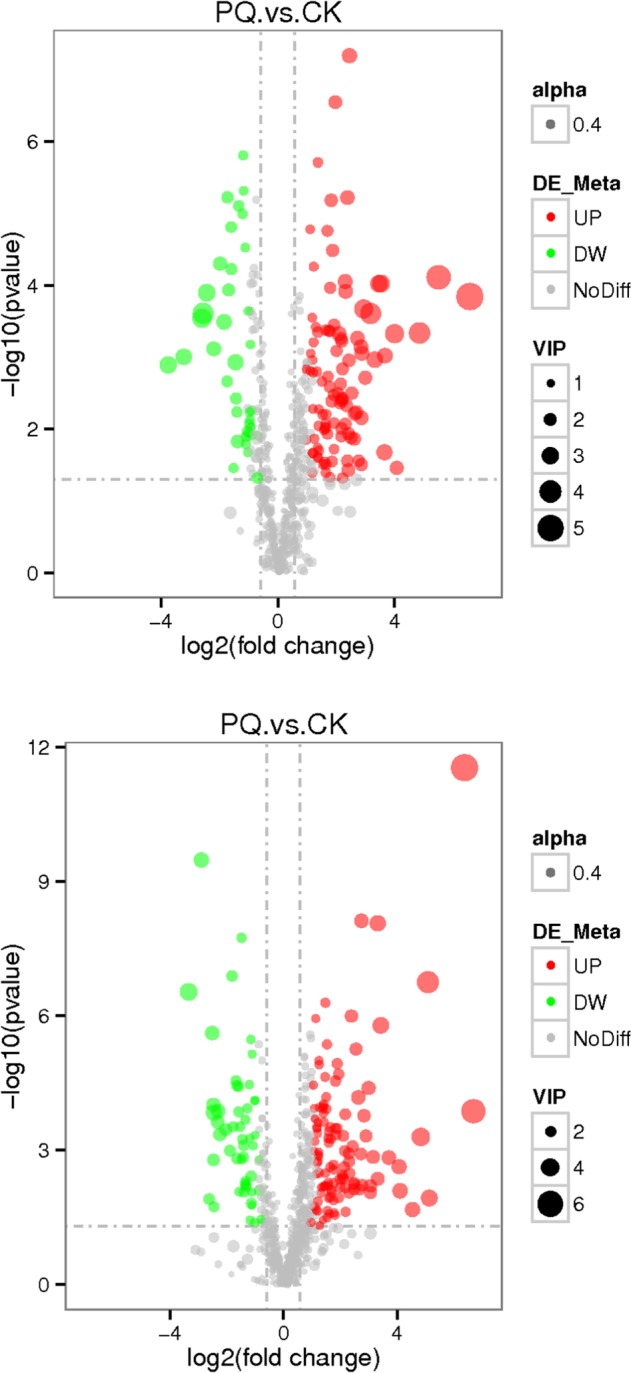
The volcano plot of the lung metabolomics in mice between the PQ poisoning group and the control group (Red represents the up-regulated metabolites compared with PQ group, green represents the down-regulated metabolites compared with PQ group, and gray represents the metabolites with no difference between the PQ group and the control group. VIP value represents the importance projection value of the metabolite obtained in the PLS-DA model compared in this group. The upper figure was obtained in the negative polarity mode, and the bottom figure was obtained in the positive polarity mode.

Metabolomics focuses on low molecular weight endogenous metabolites in biological samples^22^ and is a promising tool for identifying novel biomarkers that could help to elucidate the toxic mechanisms of PQ by investigating the changes in metabolic signatures induced by drug exposure. As shown in Fig. 4, the gray plot shows there is no difference between the PQ poisoning group and the control group. The red plots show up-regulated endogenous metabolites in the PQ group, while the green plots show down-regulated endogenous metabolites in the PQ group. The distinguished different metabolites are shown in Table 2.

**Table 2:** The significant different metabolites between the PQ group and the control group.

No.	metabolites	VIP	ROC	P value	trend	metabolic pathway
1	Mesaconic acid	1.154	0.89	0.0055	↓	Glyoxylate and dicarboxylate metabolism;
2	L-glutamic acid	1.100	1	2.99E-05	↓	Glutathione metabolism;Alanine, aspartate and glutamate metabolism;porphyrin and chlorophyll metabolism
3	Genistein	2.587	0.89	0.0012	↓	Isoflavonoid biosynthesis
4	Lumichrome	1.481	0.99	7.82E-06	↓	Riboflavin metabolism
5	Daidzein	2.441	0.90	0.0003	↓	Isoflavonoid biosynthesis
6	3-Sulfino-L-alanine	2.658	0.99	0.0001	↓	Taurine and hypotaurine metabolism
7	hypotaurine	3.105	0.94	0.0001	↓	
8	N-Acetylneuraminic acid	1.539	0.90	0.0061	↓	Amino sugar and nucleotide sugar metabolism
9	sphinganine	1.365	0.94	0.001	↓	Sphingolipid metabolism
10	testosterone	1.853	0.92	0.012	↓	Steroid degradation
11	Adenine	1.631	0.90	0.0087	↓	Purine metabolism
12	Xanthine	1.304	0.94	0.0003	↑	Purine metabolism
13	Guanine	1.064	0.86	0.0351	↓	Purine metabolism
14	Adenosine diphosphate ribose	3.271	0.93	0.00029	↓	Purine metabolism
15	Cytidine	1.122	0.88	0.0074	↓	Pyrimidine metabolism
16	Adenosine 5′-monophosphate	1.612	0.92	0.0038	↓	Pyrimidine metabolism;Regulation of lipolysis in adipocytes
17	N-Heptanoylhomoserine lactone	1.766	0.97	2.84E-05	↓	Tryptophan metabolism
18	citric acid	1.137	0.86	0.01368	↑	TAC cycle
19	N-Acetyl-L-aspartic acid	2.404	1	0.00017	↑	Alanine, aspartate and glutamate metabolism
20	glycine	1.529	0.75	0.028247109	↑	Glutathione metabolism;porphyrin and chlorophyll metabolism
21	Corticosterone	2.276	0.97	0.00047064	↑	Regulation of lipolysis in adipocytes;Aldosterone synthesis and secretion
22	desoxycortone	2.289	1	0.000478393	↑	Aldosterone synthesis and secretion
23	Cholic acid	2.003	0.78	0.037133652	↑	Primary bile acid biosynthesis
24	Prostaglandin G2	1.050	0.93	0.001097557	↑	Platelet activation
25	cinnamaldehyde	1.277	0.94	0.003255697	↑	Inflammatory mediator regulation of TRP channels
26	Arachidonic acid	1.238	0.93	0.001273429	↑	Vascular smooth muscle contraction; Platelet activation; Fc gamma R-mediated phagocytosis
27	4-Pyridoxic acid	1.736	0.93	0.002346317	↑	Vitamin B6 metabolism
28	S-(Formylmethyl)glutathione	1.264	0.79	0.046844413	↑	Metabolism of xenobiotics by cytochrome P450
29	Phenylacetylglycine	3.751	0.99	0.000505711	↑	Phenylalanine metabolism
30	2-Hydroxyphenylacetic acid	1.028	0.90	0.005251903	↑	Phenylalanine metabolism
31	Hippuric acid	1.111	0.96	0.00137572	↑	Phenylalanine metabolism
32	Vanillin	1.133	0.90	0.006734157	↑	Biosynthesis of phenylpropanoids
33	Dehydroascorbic acid	2.697	1	0.001467607	↑	Ascorbate and aldarate metabolism
34	1-methylhistidine	2.670	1	7.60E-09	↑	Histidine metabolism
35	Kynurenic acid	1.397	0.97	0.000120172	↑	Tryptophan metabolism
36	Indole-2-acetic acid	1.452	0.96	0.000375978	↑	Tryptophan metabolism
37	4-Methylphenol	4.432	1	7.72E-05	↑	Nitrotoluene degradation

#### The metabolite heatmaps in the PQ group and the control group

**Figure 5:**
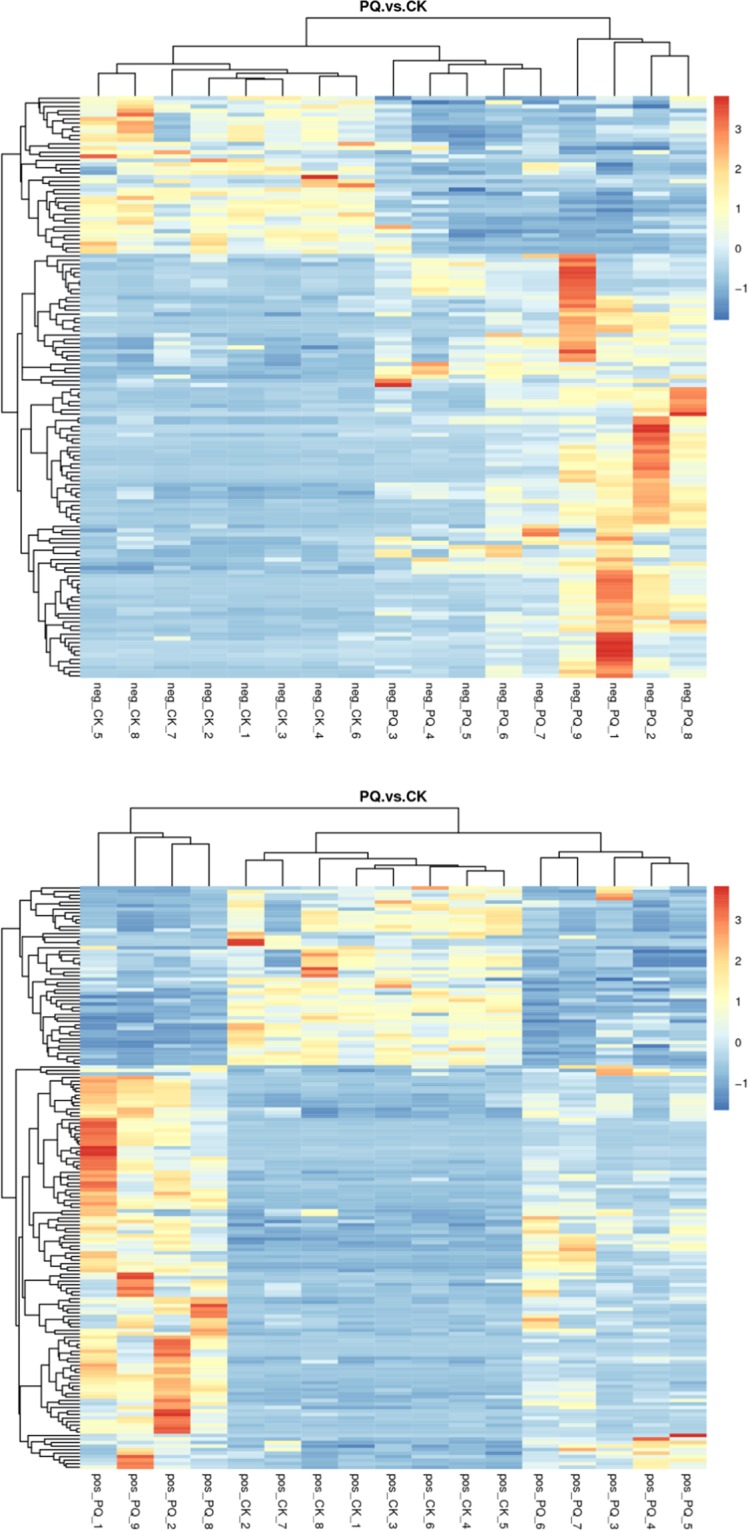
The metabolites heatmap between the PQ group and the control group (the upper figure was obtained using the negative mode, the bottom figure was obtained using the positive mode).

In order to analyze the metabolic pattern in the different groups, the metabolite heatmaps were obtained as shown in Fig. 5. There were differences in the metabolites between the PQ group and the control group.

#### The KEGG pathway

**Figure 6:**
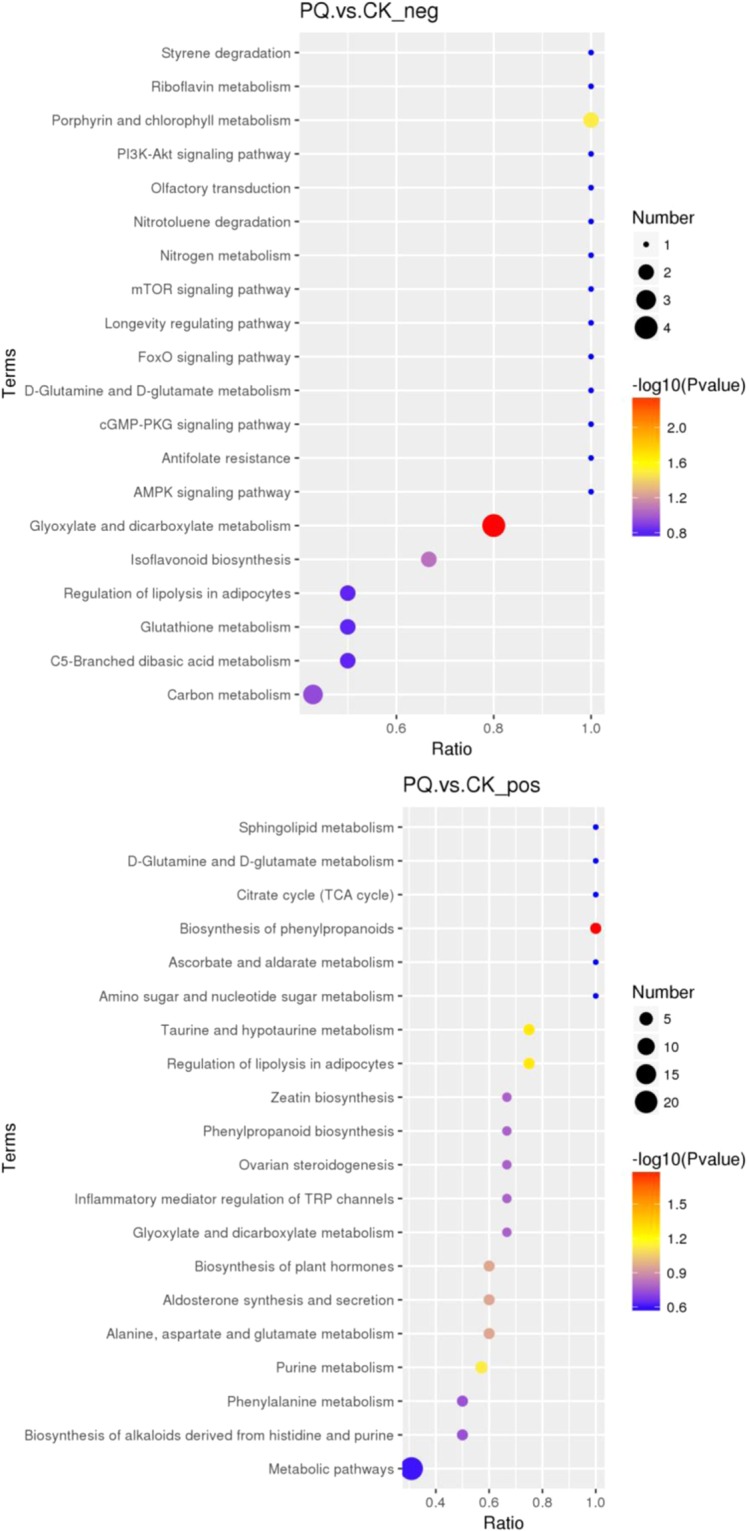
The Kegg pathway that the significant different metabolites take part in between the PQ group and the control group (the upper figure was obtained in the negative polarity mode; the bottom figure was obtained in the positive polarity mode).

In order to elucidate the toxicology of PQ, the cluster of the KEGG pathway that the distinguished different metabolites take part in was obtained as shown in Fig. 6. It can be concluded that the citrate cycle, glutathione metabolism, taurine and hypotaurine metabolism, regulation of lipolysis in adipocytes, inflammatory mediator regulation of transient receptor potential (TRP) channels, glyoxylate and dicarboxylate metabolism, alanine, aspartate and glutamate metabolism, biosynthesis of phenylpropanoids, purine and pyrimidine metabolism, aldosterone synthesis and secretion, and phenylalanine metabolism are the main metabolic pathways that reflected the toxicology of PQ poisoning.

### The metabolic pattern in the HMH group and the PQ poisoning group

#### The PCA and PLS-DA results in the PQ poisoning group and the control group

**Figure 7:**
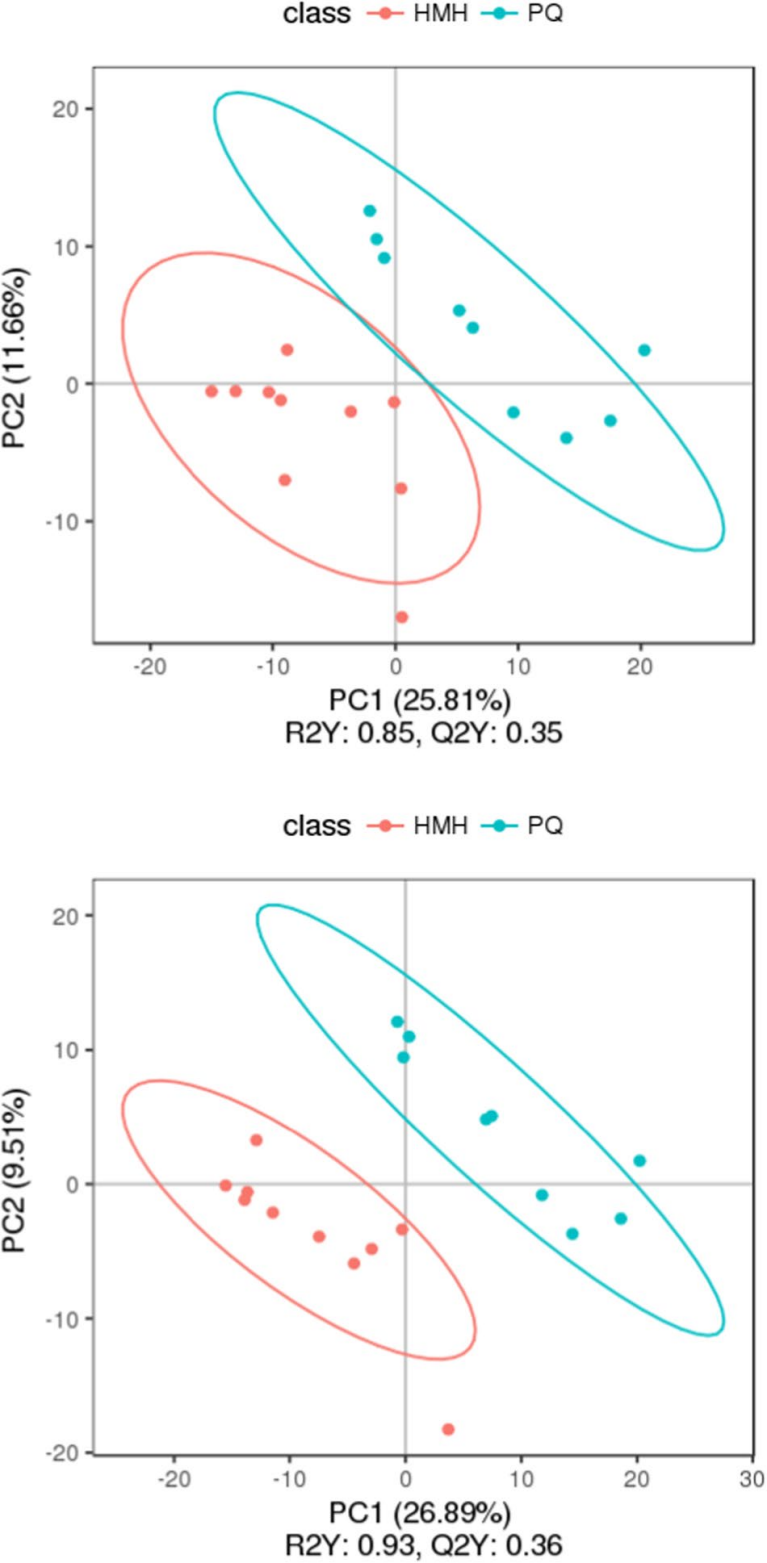
The PLS-DA, the upper figure was obtained in the negative polarity mode, the bottom figure was obtained in the positive polarity mode. Score plot from partial least squares discriminant analysis of the paraquat group and the HMH group. Each data point represents a function of the entire spectral profile of each subject (n = 19). Partial least squares discriminant analysis showed a clear separation between the 2 groups with acceptable goodness of fit (R^2^ = 0.85 or 0.93) and predictive power (Q^2^ = 0.35 or 0.36).

PCA of the metabolites in the PQ group and the HMH group provided a satisfactory separation as shown in (Supplementary Fig. 2). The potential constituents were screened by PLS-DA as shown in Fig. 7, which was used to reveal the differences between the HMH group and the PQ group. The PLS-DA scores plot showed a very good discrimination between the PQ poisoning group and the HMH group.

#### The volcano maps of differential metabolites

**Figure 8:**
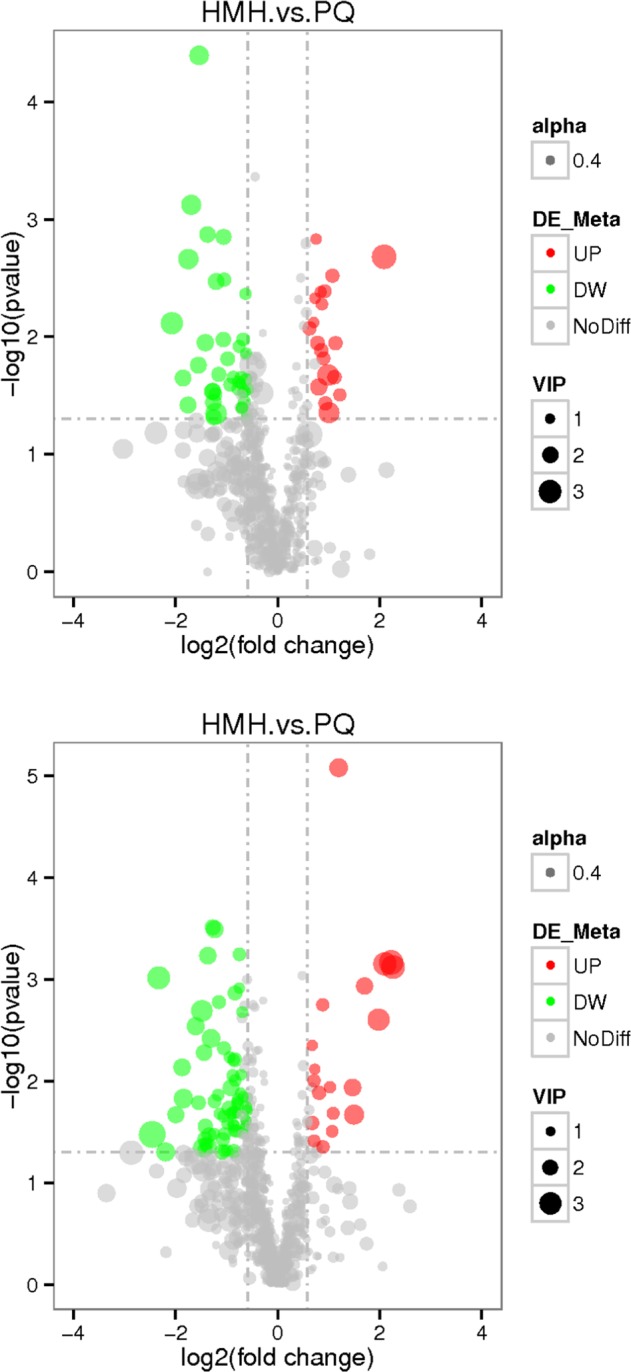
The volcano plot of the lung metabolomics in mice between the HMH and the PQ group (Red represents the up-regulated metabolites compared with PQ group, green represents the down-regulated metabolites compared with PQ group, and gray represents the metabolites with no difference between PQ group and HMH group. VIP value represents the importance projection value of the metabolite obtained in the PLS-DA model compared in this group. The upper figure was obtained in the negative polarity mode, and the bottom figure was obtained in the positive polarity mode).

As shown in Fig. 8, the gray plot shows that there was no difference between the HMH group and the PQ group. The red plots show up-regulated endogenous metabolites in the HMH group, while the green plots show down-regulated endogenous metabolites in the HMH group. The different metabolites are shown in Table 3.

**Table 3:** The significant different metabolites between the HMH and PQ group.

No.	Metabolites	VIP	ROC	P value	Trend	Metabolic pathways
1	N-acetyl-L-aspartic acid	1.77	0.88	0.0498	↑	Alanine, aspartate and glutamate metabolism
2	L-Glutamic acid	1.00	0.86	0.0075	↑	Alanine, aspartate and glutamate metabolism;Arginine biosynthesis;Histidine metabolism
3	L-Aspartic acid	1.06	0.87	0.0047	↑	Alanine, aspartate and glutamate metabolism;Arginine biosynthesis;Histidine metabolism
4	mesaconic acid	1.62	0.83	0.0113	↑	Pyrimidine metabolism;C5-Branched dibasic acid metabolism
5	Adenosine 5′-monophosphate	2.01	0.77	0.0267	↑	Regulation of lipolysis in adipocytes;Pyrimidine metabolism
6	Methylmalonic acid	1.03	0.94	0.0014	↑	Pyrimidine metabolism;Valine, leucine and isoleucine degradation
7	Cytidine	1.54	0.83	0.0368	↑	Pyrimidine metabolism
8	phosphonoacetic acid	1.25	0.86	0.0115	↑	Microbial metabolism in diverse environments;Phosphonate and phosphinate metabolism
9	Hypotaurine	2.52	0.81	0.0214	↑	Taurine and hypotaurine metabolism
10	L-Glutathione (reduced)	2.87	0.88	0.0025	↑	glutathione metabolism
11	Cysteinylglycine	3.13	0.91	0.0008	↑	glutathione metabolism
12	Succinyl proline	1.22	0.82	0.016	↓	proline metabolism
13	Corticosterone	1.25	0.8	0.0241	↓	Regulation of lipolysis in adipocytes;Aldosterone synthesis and secretion
14	Prostaglandin G2	1.5	0.89	0.0033	↓	Platelet activation
15	4-Pyridoxic acid	1.91	0.8	0.0361	↓	Vitamin B6 metabolism
16	citric acid	1.3	0.8	0.0222	↓	TCA cycle; Alanine, aspartate and glutamate metabolism
17	Xanthine	1.68	0.88	0.0062	↓	Biosynthesis of alkaloids derived from histidine and purine;purine metabolism

#### The metabolite heatmaps in the HMH group and PQ group

**Figure 9:**
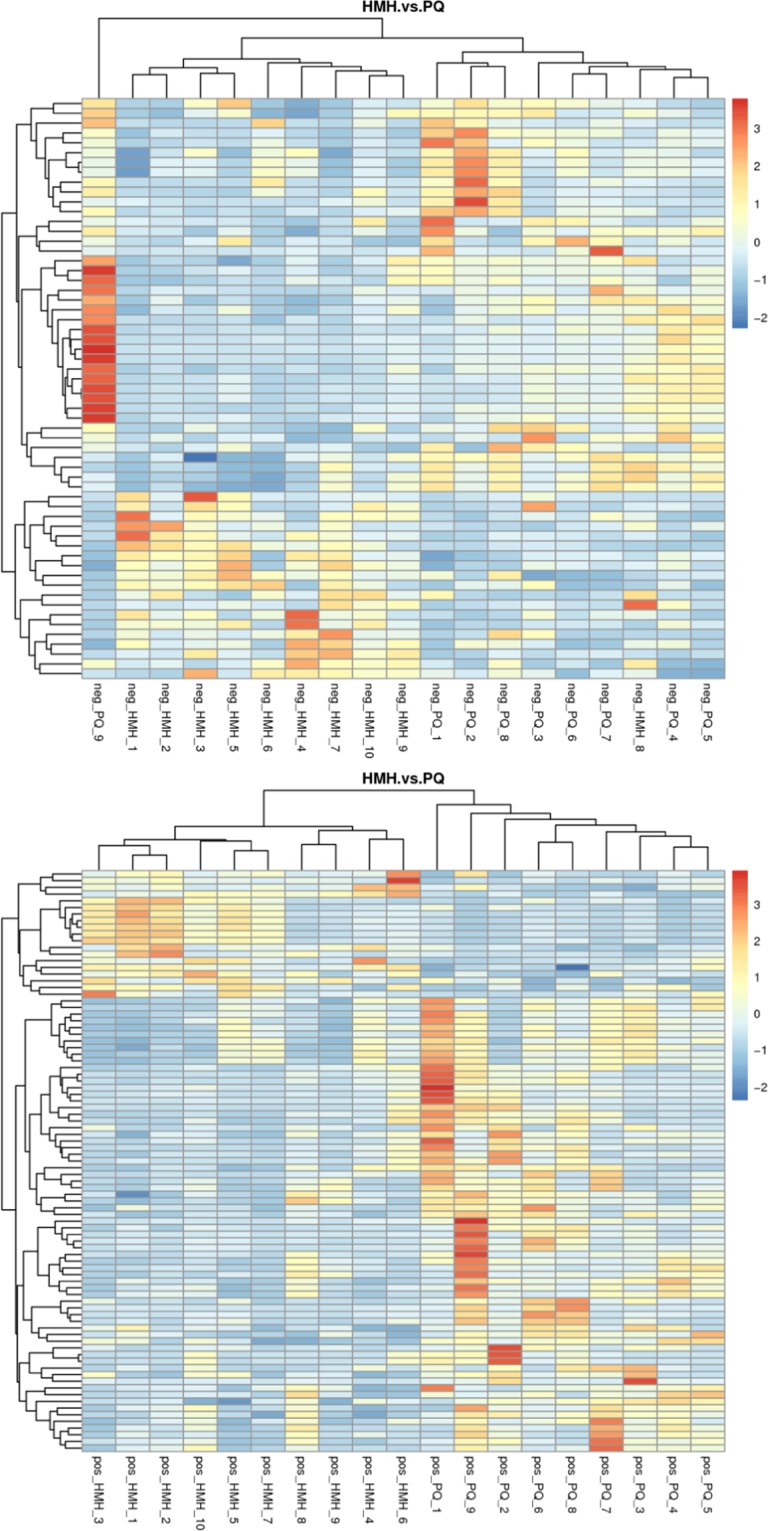
The metabolites heatmap between the HMH group and the PQ group. The upper figure means the metabolites was obtained in the negative polarity mode, the bottom figure means the metabolites was obtained in the positive polarity mode.

In order to analyze the metabolic pattern in the different groups, the metabolite heatmaps were obtained as shown in Fig. 9. There was a distinct difference in the metabolites between the HMH group and the PQ group.

#### The KEGG pathway

**Figure 10:**
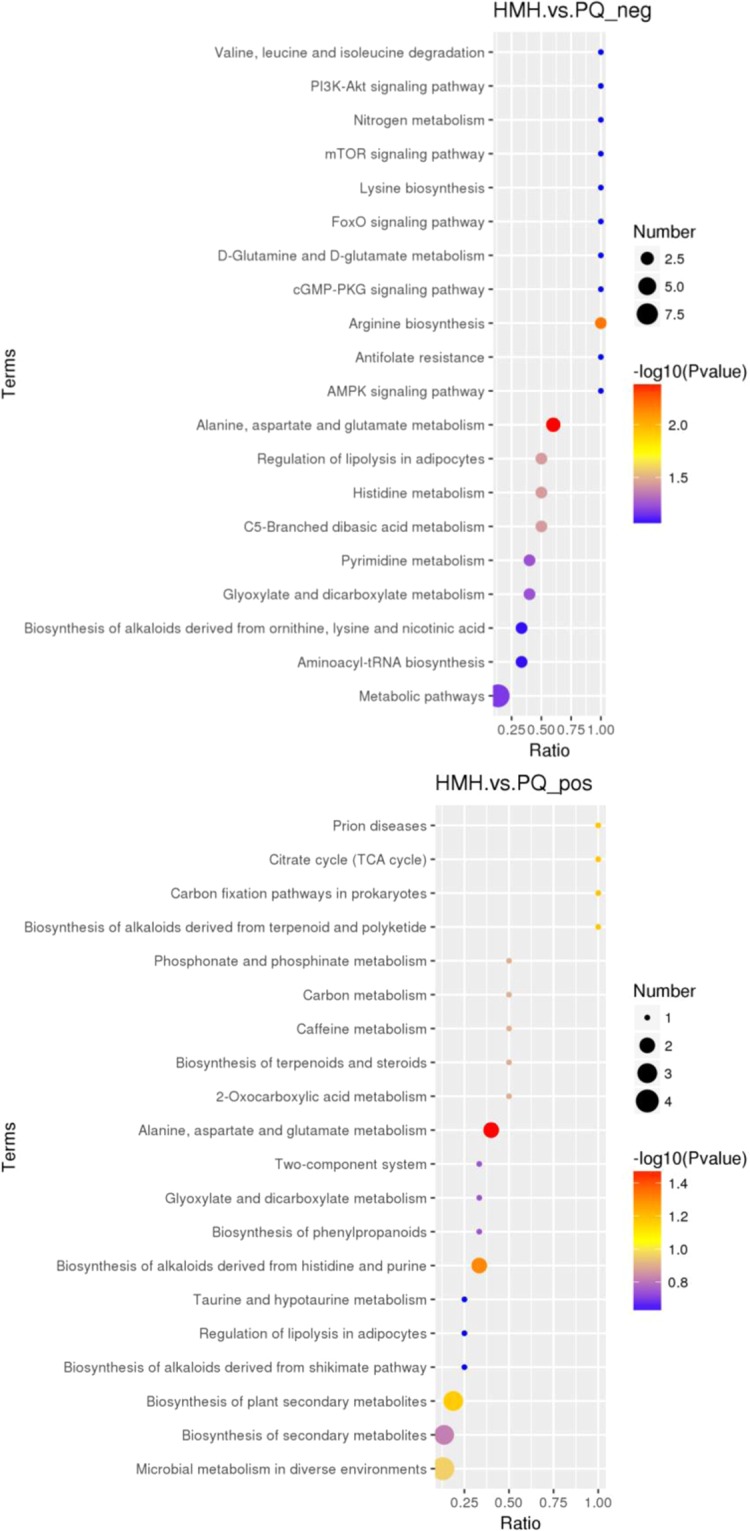
The Kegg pathway that the distinguished metabolites take part in between the HMH group and the PQ group (the upper figure was obtained in the negative polarity mode; the bottom figure was obtained in the positive polarity mode).

In order to elucidate the pharmacology of HMH, the cluster of the KEGG pathway that the distinguished different metabolites take part in was obtained as shown in Fig. 10. It can be concluded that the citrate cycle, glutathione metabolism, taurine and hypotaurine metabolism, regulation of lipolysis in adipocytes, glyoxylate and dicarboxylate metabolism, alanine, aspartate and glutamate metabolism and purine and pyrimidine metabolism are the main metabolic pathways that reflect the pharmacology of HMH treatment.

## Discussion

### The PQ toxicological mechanism

As shown in Table 1, compared to the control group, the content of MDA increased, while the activity of SOD significantly decreased in lung tissue in the PQ group. It is well known that PQ toxicity involves the generation of superoxide anions, with the subsequent formation of more toxic ROS, which result in the disruption of NADPH-requiring biochemical processes^23–25^. PQ toxicological mechanism is complex, beside the production of more extra ROS, pro-inflammatory and fibrotic cytokines are involved in lung injury caused by PQ poisoning^26^. It can be seen from Fig. 2, in the PQ group, abundant infiltration of inflammatory cells, mainly neutrophils and macrophages and a few lymphocytes, were observed in the alveolar space, accompanied by edema.

Figures 3–5 show the differences in the metabolic patterns between the PQ group and the control group. Actually, the mice in the paraquat poisoning group showed burnout and lack of activity, perhaps paraquat poisoning affect the diet in mice, that also can be reflected by the metabolomics. Because metabolomics aims to gather as much information on low-molecule metabolites in biological systems as possible, describe the actual health status of organisms.As shown in Table 2, there were the significant different metabolites between the PQ group and the control group, that mainly take part in the citrate cycle, glutathione metabolism, taurine and hypotaurine metabolism, regulation of lipolysis in adipocytes, inflammatory mediator regulation of TRP channels and purine metabolism, that can be seen from the Fig. 6. The redox cycling properties of PQ were the main mechanism in the interference of cell metabolism. A disruption in the electron transport chain and tricarboxylic acid cycle (TCA cycle) dysfunction have been found after PQ poisoning^27–29^.

As shown in Table 2, the level of adenosine 5′-monophosphate, adenine, guanine, Adenosine diphosphate ribose, cytidine and xanthine were down regulated or up regulated, which take part in the purine and pyrimidine metabolism. Compared with the control group, the levels of sphinganine and N-acetylneuraminic acid decreased significantly, indicating that PQ damaged the intact cell membrane. Moreover, the content of corticosterone increased significantly, indicating the severity of lipolysis in the cell membrane. While the level of 1-methylhistidine, prostaglandin G2, cinnamaldehyde and arachidonic acid increased in the PQ group, showing that PQ caused lung tissue inflammation.

Compared with the control group, the levels of genistein, lumichrome and daidzein decreased significantly, showing that PQ generated cumulative ROS, which consumed antioxidants in the body. The level of 4-pyridoxic acid, vanillin, and dehydroascorbic acid increased in the PQ group. 4-pyridoxic acid is a metabolite of vitamin B6, and dehydroascorbic acid is the product of ascorbic acid reacting with ROS. These compounds, including vitamin B6, ascorbic acid and vanillin are antioxidants^30^, and we speculate that organisms possess multiple endogenous defense mechanisms against ROS caused by PQ.

Compared with the control group, the level of indole-2-acetic acid, phenylacetylglycine, 2-hydroxyphenylacetic acid and hippuric acid increased, and this result is consistent with the report by Wang *et al*.^22^ and Roede *et al*.^19^. The contents of other metabolites, such as desoxycortone, corticosterone and cholic acid increased significantly, indicating that PQ affects aldosterone synthesis and secretion, and primary bile acid biosynthesis. PQ also interfered with tryptophan metabolism as the levels of N-heptanoylhomoserine lactone decreased, while the levels of kynurenic acid and indole-2-acetic acid increased significantly.

**Figure 11:**
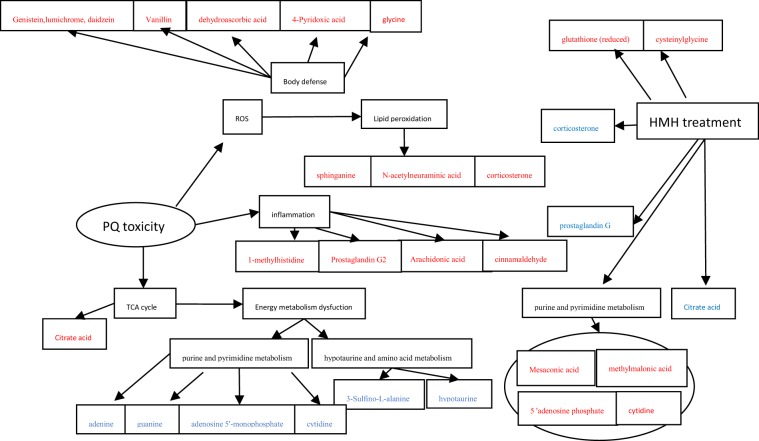
The toxicological mechanism of paraquat and the protective effect of HMH on lung injury caused by paraquat based on the metabolomics (Red represents the up-regulated metabolites compared with PQ group, blue represents the down-regulated metabolites compared with PQ group).

In conclusion, as shown in Fig. 11, PQ disrupted the electron transport chain, generating superoxide anions in mitochondria and the cytosol of mammalian cells leading to the formation of several ROS. Excessive ROS result in an imbalance of the redox state in cells causing oxidative damage including lipid peroxidation, inflammation, and finally, cell death. During this process, purine and pyrimidine metabolism, aldosterone synthesis and primary bile acid biosynthesis were interrupted. Organisms possess multiple endogenous defensive structures against ROS and oxidative stress, including vanillin, vitamin B6, ascorbic acid, SOD, and thiol-containing molecules such as glutathione^28^. Our results are in accordance with those in previous reports^19,26,31,32^. Moreover, we found more biomarkers than Wang *et al*.^22^, which helped to reveal the toxicology of PQ in more detail.

### The pharmacology of HMH

It can be seen in Fig. 2 in the HMH group, pulmonary lesions were similar to those in the PQ group, but significantly milder. The H&E staining results demonstrated that HMH alleviated the pathological changes of acute alveolitis in mice and possibly inhibited the production of hydroxyl free radicals and superoxide.

Innovations in the treatment of PQ poisoning have been focused on the use of antioxidants^32–34^. In particular, HMH is a metabolite of endogenous creatinine, and an excellent antioxidant with strong ability to eliminate hydroxyl radicals^4–8^. Compared with the PQ group, the content of MDA in the HMH group was significantly decreased, and the activity of SOD was significantly increased, suggesting that HMH has a protective effect against lung injury caused by PQ poisoning^13,14^.

As shown in Figs. 7–9, differences in the metabolic patterns between the HMH group and the PQ group were observed. Compared with the PQ group, the different metabolites was showed in Table 3. It can be seen from Fig. 10 that the significant differences in metabolites showed that HMH interfered with the alanine, aspartate and glutamate metabolism, regulation of lipolysis in adipocytes and histidine metabolism.

The contents of glutathione (reduced) and cysteinylglycine were increased to defend against damage caused by ROS. Moreover, succinyl proline, ACE inhibitors and the metabolite of proline, are also able to reduce the production of ROS in the mitochondria^35,36^. Mesaconic acid, methylmalonic acid (isosuccinic acid), 5′ adenosine phosphate, and cytosine nucleoside are all involved in the metabolism of pyrimidines *in vivo*. Compared with the PQ group, pyrimidine metabolism-related products in the HMH group were increased. Compared with the PQ group, the content of xanthine in the HMH group was lower, which take part in the purine metabolism.

Prostaglandins are lipid mediators produced by enzymatic metabolism of arachidonic acid. Compared with the PQ group, the level of prostaglandin G2 in the HMH group decreased, indicating a reduction in inflammation. Corticosterone is involved in lipid oxidation in cells, in addition to aldosterone synthesis and secretion. Decreased corticosterone content indicates decreased lipid peroxidation level and aldosterone level, which is consistent with the reported role of HMH in vascular smooth muscle^8^. Acetic acid is a short-chain fatty acid, which is involved in the synthesis of cholesterol. Other metabolites such as N-acetyl-l-aspartic acid, glutamate and hypotaurine are very good antioxidants^37,38^.

In conclusion, HMH significantly reduced the MDA level and improved SOD activity in the PQ group. As shown in Fig. 11, HMH can alleviate the inflammation, improve the lipid peroxidation injury caused by PQ and effect the energy metabolism. HMH can be used as an alternative therapy to prevent injury caused by PQ poisoning. This is the first experimental study to investigate the use of HMH in the treatment of PQ toxicity, and it is strongly recommended that HMH be administered directly after PQ poisoning. HMH may constitute an effective and promising treatment for the management of PQ poisoning. This study on the protective effect of HMH against lung injury caused by PQ poisoning had some limitations. However, based on the results of metabolomics, HMH exhibits anti-inflammatory effects, and further studies are planned to expand this work.

## Supplementary information

supplement information figure 1.

supplement information figure 2.

supplement information figure 3.
