# Supplementary material for: Toxicology of paraquat and pharmacology of the protective effect of 5-hydroxy-1-methylhydantoin on lung injury caused by paraquat based on metabolomics
Source: Sci Rep. 2020 Feb 4;10:1790. doi: 10.1038/s41598-020-58599-y (PMC7000692; doi:10.1038/s41598-020-58599-y)
Supplement: Supplementary file 1 — supplement information figure 1. [file 41598_2020_58599_MOESM1_ESM.pdf]

Toxicology of paraquat and pharmacology of the protective effect of 5-hydroxy-1-methylhydantoin on lung injury caused by paraquat based on metabolomics

Lina Gao*, Huiya Yuan, Enyu Xu, Junting Liu

(School of Forensic Medicine, China Medical University, Liaoning, China, 110014)

Pearson correlation between neg QC samples

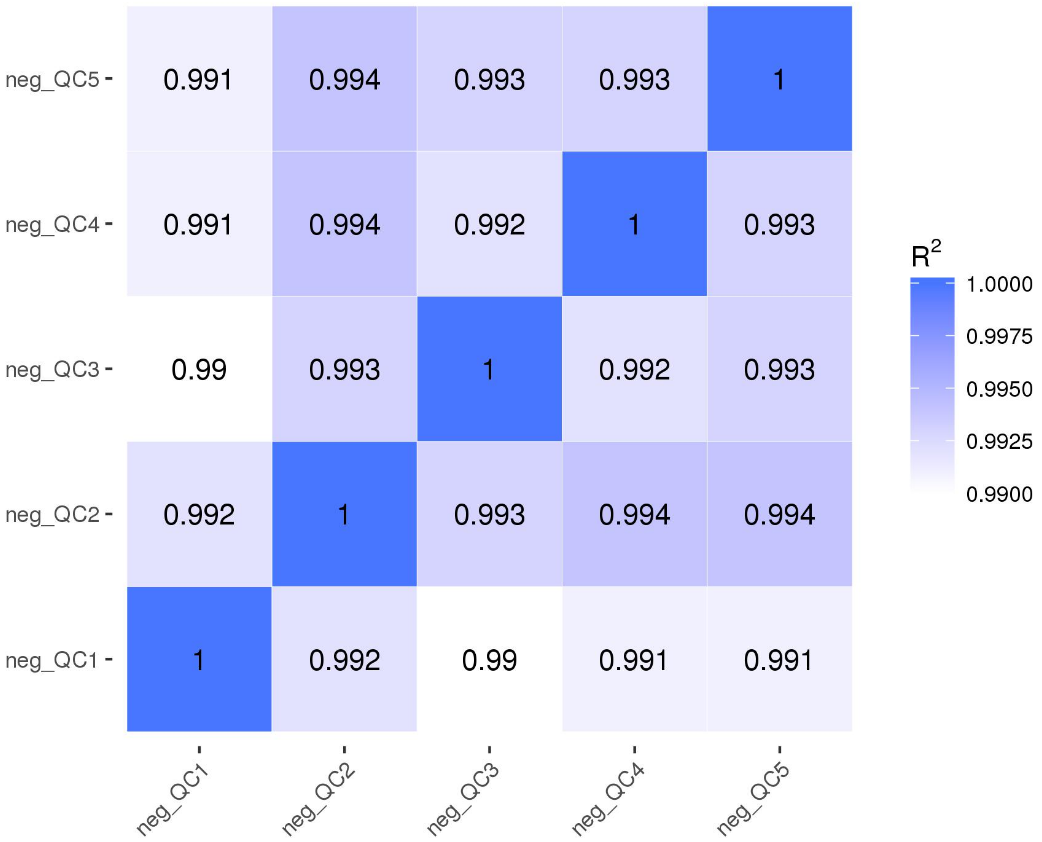

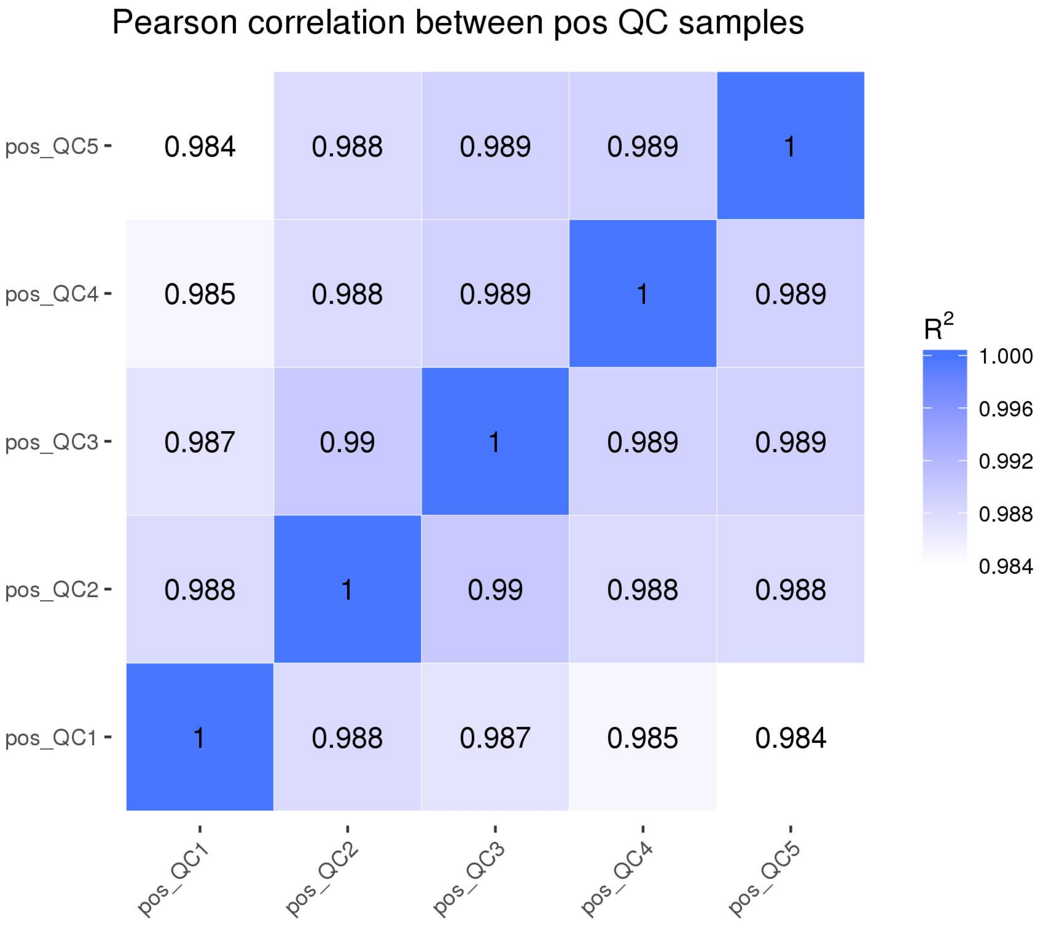

SI Fig.3. Pearson correlation between QC samples (The upper figure was obtained in the negative polarity mode; the bottom figure was obtained in the positive polarity mode).
