# Supplementary material for: Toxicology of paraquat and pharmacology of the protective effect of 5-hydroxy-1-methylhydantoin on lung injury caused by paraquat based on metabolomics
Source: Sci Rep. 2020 Feb 4;10:1790. doi: 10.1038/s41598-020-58599-y (PMC7000692; doi:10.1038/s41598-020-58599-y)
Supplement: Supplementary file 2 — supplement information figure 2. [file 41598_2020_58599_MOESM2_ESM.pdf]

Toxicology of paraquat and pharmacology of the protective effect of 5-hydroxy-1-methylhydantoin on lung injury caused by paraquat based on metabolomics

Lina Gao*, Huiya Yuan, Enyu Xu, Junting Liu

(School of Forensic Medicine, China Medical University, Liaoning, China, 110014)

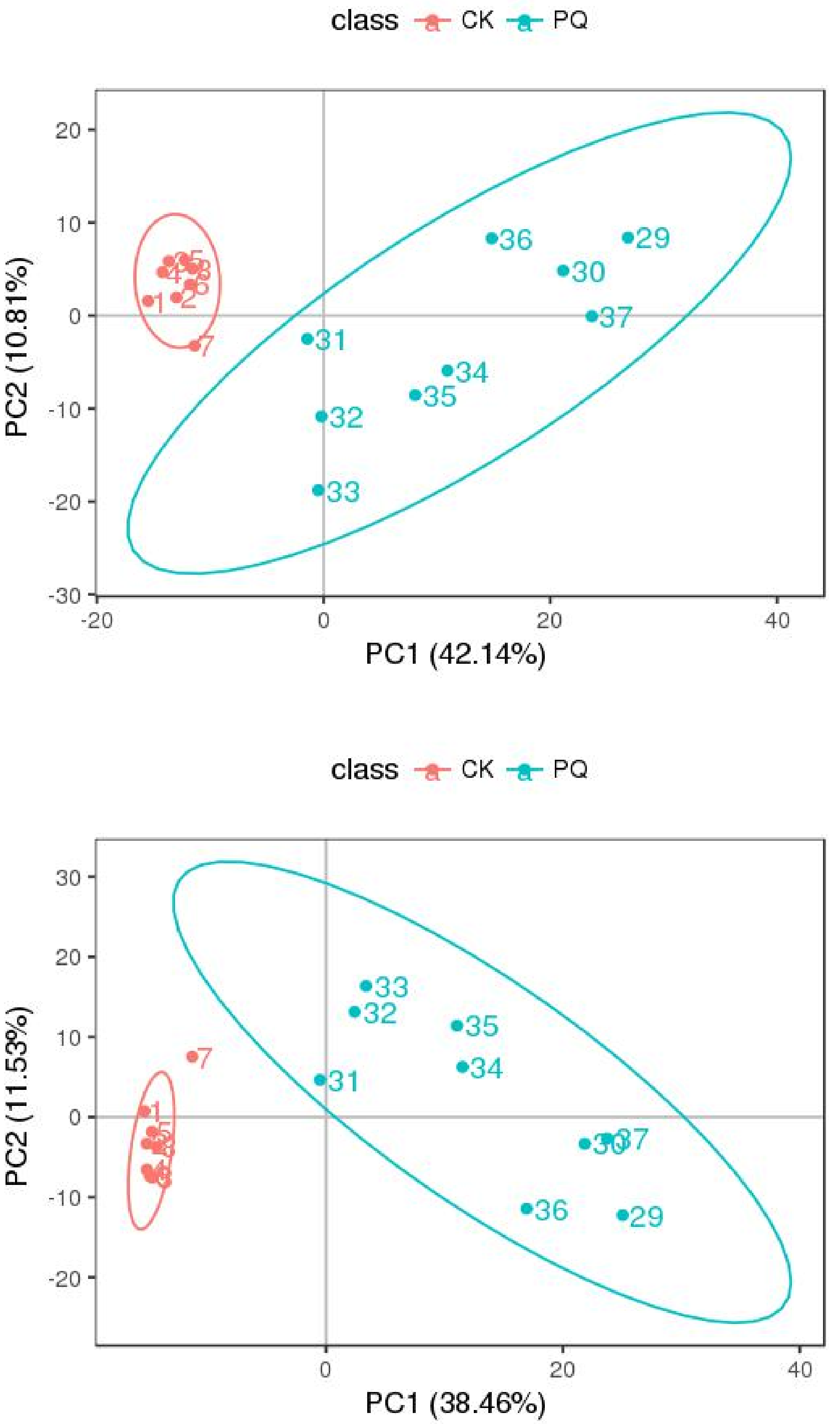

SI Fig.1. Principal component analysis between the PQ group and the control group, the upper figure was obtained in the negative mode, the bottom figure was obtained in the positive mode.
