# Supplementary material for: Toxicology of paraquat and pharmacology of the protective effect of 5-hydroxy-1-methylhydantoin on lung injury caused by paraquat based on metabolomics
Source: Sci Rep. 2020 Feb 4;10:1790. doi: 10.1038/s41598-020-58599-y (PMC7000692; doi:10.1038/s41598-020-58599-y)
Supplement: Supplementary file 3 — supplement information figure 3. [file 41598_2020_58599_MOESM3_ESM.pdf]

Toxicology of paraquat and pharmacology of the protective effect of 5-hydroxy-1-methylhydantoin on lung injury caused by paraquat based on metabolomics

Lina Gao*, Huiya Yuan, Enyu Xu, Junting Liu

(School of Forensic Medicine, China Medical University, Liaoning, China, 110014)

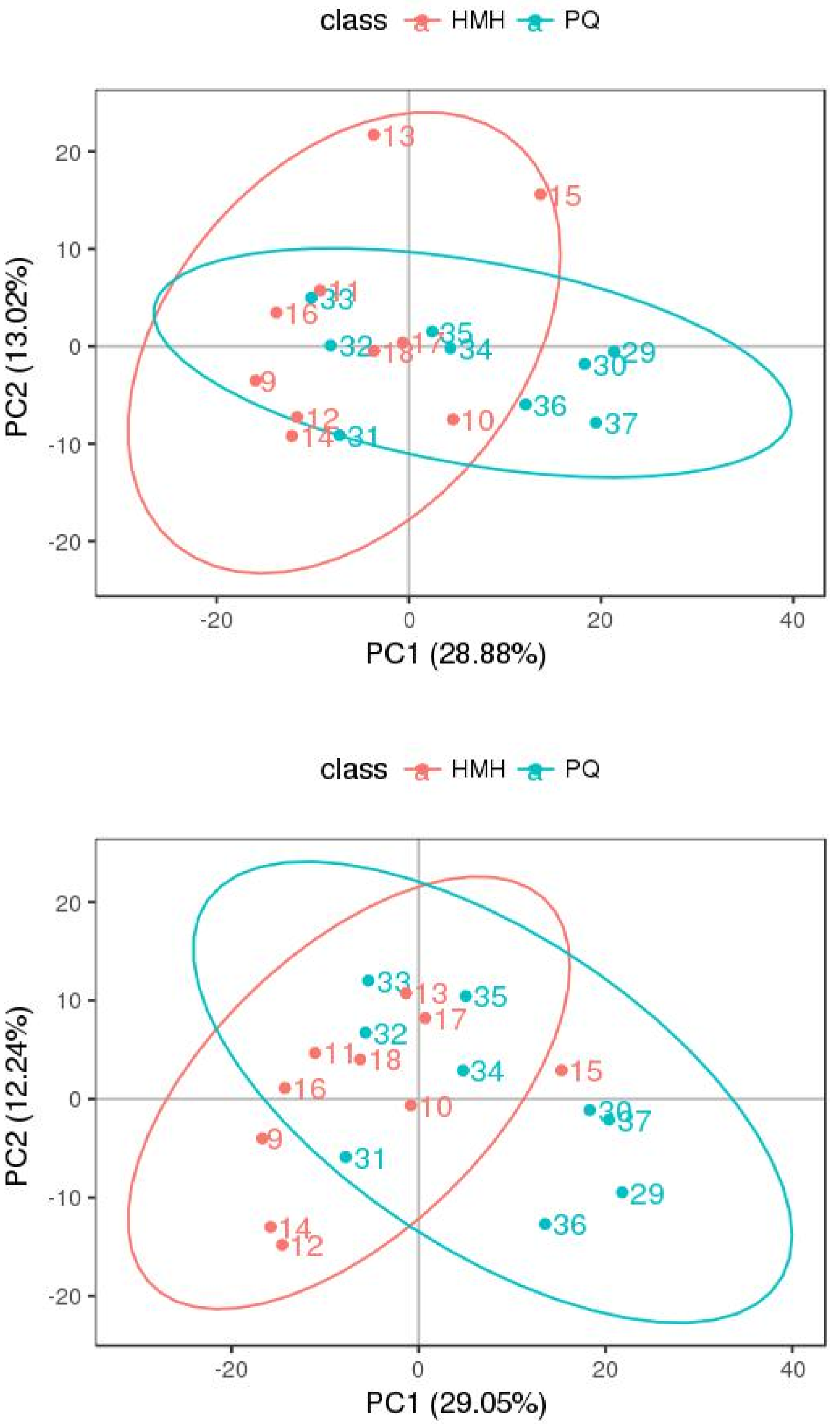

SI Fig.2. The principal component analysis between the HMH group and the PQ group (The upper figure was obtained in the negative polarity mode; the bottom figure was obtained in the positive polarity mode).
